# ULSL: Unified Latent and Similarity Learning for robust multi-omics cancer subtype identification

**DOI:** 10.1093/bioadv/vbag110

**Published:** 2026-04-17

**Authors:** Zhiyong Liu, Yuhao Zhou, Wenqing Yang, Yashu Zhang, Yan He, Donghan Li, Songming Zheng, Shiqi Chen, Lijun Fan

**Affiliations:** Center for Endemic Disease Control, Chinese Center for Disease Control and Prevention, Harbin Medical University, Harbin, 150086, China; Center for Endemic Disease Control, Chinese Center for Disease Control and Prevention, Harbin Medical University, Harbin, 150086, China; Center for Endemic Disease Control, Chinese Center for Disease Control and Prevention, Harbin Medical University, Harbin, 150086, China; Center for Endemic Disease Control, Chinese Center for Disease Control and Prevention, Harbin Medical University, Harbin, 150086, China; Center for Endemic Disease Control, Chinese Center for Disease Control and Prevention, Harbin Medical University, Harbin, 150086, China; Center for Endemic Disease Control, Chinese Center for Disease Control and Prevention, Harbin Medical University, Harbin, 150086, China; Center for Endemic Disease Control, Chinese Center for Disease Control and Prevention, Harbin Medical University, Harbin, 150086, China; Center for Endemic Disease Control, Chinese Center for Disease Control and Prevention, Harbin Medical University, Harbin, 150086, China; Key Laboratory of Etiology and Epidemiology (23618504), National Health Commission, Education Bureau of Heilongjiang Province, Harbin, 150086, China; Heilongjiang Provincial Key Laboratory of Trace Elements and Human Health, Harbin Medical University, Harbin, 150086, China

## Abstract

**Motivation:**

Cancer’s high heterogeneity necessitates precise molecular classification for improved clinical outcomes. However, current multi-omics clustering often struggles with molecular complexity. We propose Unified Latent and Similarity Learning (ULSL), a novel framework that simultaneously learns latent embeddings and similarity matrices through unified optimization. ULSL employs graph fusion for cross-omics structural consistency and latent representation learning to project data into low-dimensional spaces, effectively mitigating noise and high dimensionality.

**Results:**

ULSL was evaluated on synthetic datasets and 10 public cancer datasets from The Cancer Genome Atlas (TCGA). It consistently outperformed seven state-of-the-art methods in accuracy and robustness for subtype identification. On simulated datasets, ULSL maintained superior performance even with weak signal features and high noise levels. On TCGA datasets, ULSL not only identified survival-associated subtypes in a larger number of cancer types but also detected a greater number of clinically enriched features compared to competing approaches. Furthermore, the specific case study on AML demonstrated that ULSL aligns with the biological basis of the traditional FAB classification while offering distinct advantages in prognostic stratification.

**Availability and implementation:**

The source code for ULSL is available at https://github.com/codelzy-01/ULSL-1.git

## 1 Introduction

Cancer is a highly heterogeneous and complex disease, with substantial variation in clinical presentation and therapeutic response (Proietto *et al.* 2023). This diversity is primarily reflected in metastatic potential, prognostic features, and treatment sensitivity (Fisher *et al.* 2013; Dagogo-Jack and Shaw 2018; Zhang *et al.* 2022). With the advancement of precision medicine, the accurate identification and classification of cancer subtypes have become critical for both clinical practice and biomedical research. Traditionally, cancer classification has been based on anatomical location and histopathological characteristics; however, such approaches fail to fully capture the molecular essence of the disease. Recent breakthroughs in high-throughput sequencing technologies and multi-omics profiling have provided novel perspectives for cancer subtyping. Early molecular classification studies often focused on a single omics layer (genomics or transcriptomics) (Cgar 2012, 2014; Tang *et al.* 2020). While these approaches revealed important tumor features within specific molecular dimensions, they were insufficient to comprehensively capture the biological complexity of cancer (Ritchie *et al.* 2015). To better understand the intricate regulatory mechanisms underlying tumor development, researchers have increasingly turned to integrative multi-omics analyses for molecular subtyping (Cai *et al.* 2022; He *et al.* 2023). This systems biology approach enables a more holistic characterization of molecular features and biological behaviors, thereby facilitating precise identification of cancer subtypes and the development of personalized therapeutic strategies tailored to subtype-specific characteristics.

In recent years, a variety of multi-omics clustering algorithms have been developed for cancer molecular subtype analysis. Similarity network fusion (SNF) (Wang *et al.* 2014) constructs similarity networks for each omics dataset based on Gaussian distance and neighborhood relationships, iteratively fuses these networks through a message-passing mechanism, and finally applies spectral clustering for partitioning. Neighborhood based Multi-Omics clustering (NEMO) (Rappoport and Shamir 2019) defines relative similarity matrices using radial basis function kernels for each dataset and integrates them via simple averaging. iClusterBayes (Mo *et al.* 2018) employs Bayesian variable selection to learn latent variables, thereby capturing the intrinsic structure of multi-omics data. MOSD (Duan *et al.* 2024) first builds locally scaled affinity matrices, then integrates them through weighted linear combination, and further enhances patient similarity via self-diffusion to facilitate clustering. CIMLR (Ramazzotti *et al.* 2018) learns sample-to-sample similarity measures by combining multiple Gaussian kernels, enforces a block structure in the resulting similarity matrix, and subsequently applies dimensionality reduction and k-means clustering. In addition, Multi-omics clustering method based on latent sub-space learning (MCLS) (Ye *et al.* 2023) leverages principal components analysis (PCA) and singular value decomposition (SVD) to construct a latent subspace, projects incomplete multi-omics data into this subspace, and performs spectral clustering, thus effectively handling missing data. Graph-driven Deep Multi-view Clustering (GDMVC) (Bai *et al.* 2024) introduces a deep learning perspective by utilizing adaptive graph autoencoders (AdaGAE) to capture view-specific topological structures. It employs a self-paced learning mechanism to dynamically refine the graph structure during training and integrates multi-view representations through a consensus constraint, effectively fusing heterogeneous omics data for clustering tasks. SCMVC (Wu *et al.* 2024) is also a deep multi-view clustering method that decouples consistency and reconstruction objectives by establishing a hierarchical feature fusion framework, and utilizes a self-weighted contrastive fusion mechanism to adaptively enhance effective views while suppressing unreliable ones. These approaches, each with distinct characteristics, provide diverse solutions for multi-omics integration and cancer subtype analysis.

Despite encouraging progress, several challenges remain unresolved. First, most existing multi-view spectral clustering methods require pre-construction of similarity matrices, which are rarely updated during optimization. Clustering performance is often overly dependent on the quality of these predefined matrices (Speicher and Pfeifer 2015; Ma and Zhang 2018; Ramazzotti *et al.* 2018; Yu *et al.* 2019). Second, omics data are characterized by a small number of samples coupled with extremely high-dimensional features, often accompanied by substantial noise and redundancy. The high dimensionality can significantly compromise clustering performance (Palsson and Zengler 2010). Although feature selection methods can partially alleviate noise, they carry the risk of discarding important patient-specific information. Third, another category of methods integrates data solely at the similarity level, such as NEMO (Rappoport and Shamir 2019), which constructs similarity networks for each omics and then fuses them. Although these strategies partially alleviate the problems caused by feature concatenation, their integration schemes often rely on coarse direct weighting or predefined rules, lacking explicit modeling of the latent complementarity among omics. As a result, they struggle to effectively capture the underlying structural patterns across datasets. Despite the rapid evolution of deep learning, its application in omics analysis faces substantial challenges. These models introduce numerous sensitive hyperparameters that lead to performance instability (Bai *et al.* 2024; Wu *et al.* 2024). Furthermore, their inherent reliance on large-scale training datasets fundamentally conflicts with the sample scarcity characteristic of omics studies (Moon and Lee 2022; Qiu *et al.* 2025). This misalignment exacerbates the risk of overfitting, thereby severely compromising their robustness and practical utility in clinical scenarios.

To address these challenges, we propose a novel method called Unified Latent and Similarity Learning (ULSL), which jointly learns latent embeddings and a fused sample similarity matrix within a unified framework and leverages their intrinsic interdependence for iterative updating and integration. The graph fusion module enhances structural consistency across omics layers by iteratively updating sample similarity networks, causing relationships among samples in different omics to gradually converge, effectively capturing cross-omics consistent structures while integrating both shared and complementary information, resulting in a more comprehensive representation. The latent representation learning module emphasizes the commonalities of samples across views by projecting each omics dataset into a low-dimensional space, effectively mitigating noise and the curse of dimensionality. By combining these two modules, ULSL achieves a dual-level integration of structural and feature information, enabling more effective extraction and fusion of relevant signals from heterogeneous multi-omics data and ultimately improving the accuracy of cancer subtype discovery.

## 2 Methods

In this paper, matrices are denoted by uppercase letters (e.g.X), vectors by bold lowercase letters (e.g.x), and scalars by lowercase letters (e.g.x). For a matrix X, $x_{ij}$ denotes the entry in the i-th row and j-th column, and $x_{j}$ represents its j-th column vector. The transpose and trace of matrix X are denoted by $X^{T}$ and Tr(X), respectively. The Frobenius norm is represented by$|\cdot {|}_{F}$. The identity matrix is denoted by I, and 1 denotes a column vector with all elements equal to one.

### 2.1 Latent representation learning

To effectively integrate heterogeneous multi-omics data while reducing noise and redundancy, we first aim to learn a shared latent representation that captures the intrinsic structure of samples across different omics views.

Inspired by Chen *et al.* (2020), we assume that each sample is associated with a latent representation that fundamentally characterizes its intrinsic features, while the different omics datasets can be regarded as observations of this latent representation from distinct perspectives. Let n be the number of samples and t be the number of views. We define the shared latent representation matrix as $H\in R^{d\times n}$, where d is the dimension of the latent subspace. For a multi-omics dataset comprising t distinct types, let $X^{\left(1\right)},X^{\left(2\right)},\ldots ,X^{\left(v\right)},\ldots ,X^{\left(t\right)}$ represent the data matrices for these t omics views. The observed data for the v-th view, denoted by $X^{\left(v\right)}\in R^{d^{\left(v\right)}\times n}$, is obtained by projecting this latent representation via a view-specific matrix $W^{\left(v\right)}\in R^{d^{\left(v\right)}\times d}$, Mathematically, this relationship is modeled as $X^{\left(v\right)}=W^{\left(v\right)}H$. Specifically, given a multi-view dataset $X=\{X^{\left(v\right)}{\}}_{v=1}^{t}$, which contains n samples and V different omics views, one can recover the shared latent representation of each data point across the different omics by means of the projection spaces $W=\{W^{\left(v\right)}{\}}_{v=1}^{t}$. This allows us to extract the consistency information embedded within all omics data of the samples. To obtain H, the problem can be formulated as solving the following optimization task:


(1) $$\begin{array}{c} \underset{W^{\left(v\right)},H}{\min}\sum_{v=1}^{t}\|X^{\left(v\right)}-W^{\left(v\right)}H{\|}_{F}^{2}\\ s.t.\;\|w_{j}{\;}^{\left(v\right)}{\|}_{2}^{2}\leq 1. \end{array}$$

The learned latent representation provides a compact and noise-reduced description of each sample, which will be further exploited to guide the construction and refinement of sample similarity relationships in the subsequent graph-based integration stage.

### 2.2 Similarity network fusion

Similarity Network Fusion (SNF) (Wang *et al.* 2014) is a widely used method for integrating multi-omics or multi-view data by constructing and iteratively fusing sample similarity networks, and it is employed in this work to provide an initial fused similarity structure. Suppose there are n samples, with their pairwise similarities represented by a matrix $E^{\left(v\right)}\in R^{n\times n}$, where $e_{ij}$ quantifies the similarity between samples $x_{i}$ and $x_{j}$. A commonly used similarity measure is a scaled exponential kernel:


(2) $$e_{ij}=\text{exp}\;\left(-\frac{\rho_{ij}{\;}^{2}}{\mu \varepsilon_{ij}}\right)$$

where $\rho_{ij}$ is the Euclidean distance between samples $x_{i}$ and $x_{j}$, μ is an empirical hyperparameter, and $\varepsilon_{ij}$ is computed from the average distances of $x_{i}$ and $x_{j}$ to their neighbors to mitigate scaling issues.

After constructing similarity matrices for each omics, SNF normalizes them to produce a global kernel matrix P and a local kernel matrix S. The entries of these matrices are defined as:


pij={eij2Σk≠ieik,j≠i   1/2,j=i  and sij={eijΣk∈N(i)eik,j∈N(i)    0otherwise .

where $N^{\left(i\right)}$ denotes the set of neighbors of $x_{i}$, including itself. Subsequently, the algorithm iteratively updates the similarity networks through message passing according to the formula:


(3) $$P^{\left(\nu \right)}=S^{\left(\nu \right)}\left(\frac{\Sigma_{k\neq \nu }P^{\left(k\right)}}{t-1}\right){\left(S^{\left(\nu \right)}\right)}^{T},\nu =1,2,\ldots ,t,$$

and fuses the networks across different omics views. This process ultimately converges to a single unified similarity network A, which captures the shared structural information among samples across all omics types, while preserving the local structural features of each individual omics and enhancing cross-omics consistency.


(4) $$A=\frac{\sum_{v=1}^{t}P^{\left(v\right)}}{t}$$

In this study, the SNF-derived fused network is used as a reference graph that provides prior structural information for subsequent similarity graph refinement.

### 2.3 Guided update of the fused similarity graph

Although SNF provides an effective way to integrate multi-omics similarity information, the resulting fused graph is obtained in a standalone manner and is not explicitly coupled with the learned latent representation. Moreover, similarity networks constructed in advance may contain noise or view-specific bias. To address these limitations, we further introduce a fused similarity graph that can be iteratively refined under the guidance of latent representations.

Specifically, we denote by $Z\in R^{n\times n}$ the fused similarity graph to be learned, where each entry $Z_{ij}$ represents the similarity between samples i and j in the integrated multi-omics space.

Specifically, the optimization of Z is achieved by introducing the following constraint term:


(5) $$\begin{array}{c} \underset{Z}{\min}\mathrm{||}Z-A{\mathrm{||}}_{F}^{2}+\beta Tr(HL_{Z}H^{T})\\ s.t.\;0\leq z_{ij}\leq 1,{\left(z_{i}\right)}^{T}1=1 \end{array}$$

A is a pre-generated reference graph obtained from Similarity Network Fusion. In the first term of Equation (6), we incorporate the discrepancy between the graph Z to be optimized and the reference structure A into the overall objective function as a penalty term. By minimizing their Frobenius norm, this term drives Z closer to A during optimization, thereby preserving the existing structural information and avoiding structural deviation, which reflects the effective utilization of the fused graph structure. In the second term, a local constraint term $Tr(HL_{Z}H^{T})$ is introduced, whereby the updating of the similarity matrix is guided by the latent representation matrix. This encourages the similarity matrix Z to learn neighborhood relationships between samples from the latent representation. Here, the Laplacian matrix is denoted as $L_{Z}=D-\frac{\left(Z^{T}+Z\right)}{2}$, the degree matrix D is a diagonal matrix $d_{ii}=\sum_{j=1}^{n}\frac{z_{ij}+z_{ji}}{2}$, By minimizing $Tr(HL_{Z}H^{T})$, nearby data points in the latent space are encouraged to exhibit higher similarity in the learned graph structure space.

### 2.4 Unified Latent and Similarity Learning (ULSL)

As mentioned above, Equations (1) and (5) can be combined into the final objective function, namely the proposed ULSL, which can be expressed as follows:


(6) $$\begin{array}{c} \underset{W^{\left(v\right)},H,Z}{\min}\sum_{v=1}^{t}\mathrm{||}X^{\left(v\right)}-W^{\left(v\right)}H{\mathrm{||}}_{F}^{2}+\alpha \mathrm{||}Z-A{\mathrm{||}}_{F}^{2}+\beta Tr(HL_{Z}H^{T})\\ s.t.\mathrm{||}w_{j}{\;}^{\left(v\right)}{\mathrm{||}}_{2}^{2}\leq 1,0\leq z_{ij}\leq 1,{\left(z_{i}\right)}^{T}1=1 \end{array}$$

Here, α>0 and β>0 are regularization parameters that balance the three terms in the objective function. The first term aims to extract the latent representation matrix H and reconstruct the observed data through each projection matrix $W^{\left(v\right)}$. The second term guides the updating of the fused similarity graph Z, enforcing Z to remain close to the prior similarity matrix A, thereby preserving existing structural information and preventing structural deviation. The final term penalizes deviations of the fused similarity graph Z from the local similarity structure implied by the latent representation H during iterative updates. Latent representation learning projects the multi-omics data into a low-dimensional space, effectively removing noise and capturing the consistent features across omics layers. By dynamically updating the fused similarity graph Z rather than relying on a fixed pre-constructed matrix, this approach addresses the common issue in traditional multi-view spectral clustering methods where clustering performance is overly sensitive to the quality of predefined similarity matrices. Within the entire framework, H guides the structural learning of Z, while Z, through the graph regularization term, constrains the geometric consistency of H, establishing a bi-directional feedback mechanism between latent representation learning and similarity graph optimization.

### 2.5 Algorithm optimization

Since the variables in Problem (6) are interdependent, the solution can be obtained through an alternating iterative optimization strategy. The detailed procedure is as follows:

1. Fix H and Z, and update W. In this case, Problem (8) reduces to:


(7) $$\underset{W^{\left(v\right)}}{\min}\sum_{v=1}^{t}\mathrm{||}X^{\left(v\right)}-W^{\left(v\right)}H{\mathrm{||}}_{F}^{2},s.t.\mathrm{||}w_{j}{\;}^{\left(v\right)}{\mathrm{||}}_{2}^{2}\leq 1.$$

In Equation (9), the optimization for each omics dataset is independent. Therefore, $W^{\left(v\right)}$ can be updated separately for each omics dataset.


(8) $$\begin{array}{c} \underset{W^{\left(v\right)}}{\min}\mathrm{||}X^{\left(v\right)}-W^{\left(v\right)}H{\mathrm{||}}_{F}^{2}\\ s.t.\mathrm{||}w_{j}{\;}^{\left(v\right)}{\mathrm{||}}_{2}^{2}\leq 1 \end{array}$$

This problem can be optimized by introducing an auxiliary variable G.


(9) $$\underset{W^{\left(v\right)},G}{\min}\mathrm{||}X^{\left(v\right)}-W^{\left(v\right)}H{\mathrm{||}}_{F}^{2},\;s.t.\;W^{\left(v\right)}=G\;,\;\mathrm{||}g_{j}{\mathrm{||}}_{2}^{2}\leq 1$$

In this way, the optimal solution to Problem (9) can be obtained using the Alternating Direction Method of Multipliers (ADMM) algorithm (Gu *et al.* 2014).


(10) $$\{\begin{array}{c} {\left(W^{\left(v\right)}\right)}^{r+1}=\arg\underset{W^{\left(v\right)}}{\min}\mathrm{||}X^{\left(v\right)}-W^{\left(v\right)}H{\mathrm{||}}_{F}^{2}+\rho \mathrm{||}W^{\left(v\right)}-G^{r}+T^{r}{\mathrm{||}}_{F}^{2},\\ G^{r+1}=\arg\underset{G}{\min}\rho \mathrm{||}{\left(W^{\left(v\right)}\right)}^{r+1}-G+T^{r}{\mathrm{||}}_{F}^{2},\;s.t.\;\mathrm{||}g_{j}{\mathrm{||}}_{2}^{2}\leq 1,\\ T^{r+1}=T^{r}+{\left(W^{\left(v\right)}\right)}^{r+1}-G^{r+1}, \end{array}$$

Here, r denotes the iteration step, and T is introduced as an intermediate variable. Finally, $W^{\left(v\right)}$can be efficiently optimized to converge to a suitable solution via ADMM.

2. Fix W and Z, update H. When W and Z are fixed, the objective function (8) can be equivalently written as:


(11) $$\underset{H}{\min}Tr\left(H^{T}EH\right)-2Tr\left(H^{T}Q\right)+\beta Tr(HL_{Z}H^{T})$$

where by taking the partial derivative of the objective function with respect to H and setting it equal to zero, we derive the following optimality condition:


(12) $$E^{T}H-Q+\beta HL_{Z}=0$$

Equation (12) is a standard Sylvester equation, which can be solved using the Bartels–Stewart algorithm (Bartels and Stewart 1972; Ge *et al.* 2023). To ensure numerical stability during the solution process, we set $\hat{L_{Z}}=L_{Z}+\epsilon I$, where ϵ is an arbitrarily small positive constant.

3. Fix H and W, update Z. the objective function (8) can be equivalently written as:


(13) $$\begin{array}{c} \underset{z_{ij}}{\min}\sum_{i=1}^{n}\sum_{j=1}^{n}(\alpha (z_{ij}-a_{ij}{)}^{2}+\beta \mathrm{||}h_{i}-h_{j}{\mathrm{||}}_{2}^{2}z_{ij})\\ s.t.\;0\leq z_{ij}\leq 1,{\left(z_{i}\right)}^{T}1=1 \end{array}$$

The problem in (13) can be decomposed into n subproblems. By setting $d_{ij}=\beta |h_{i}-h_{j}{|}_{2}^{2}$, $c_{ij}=a_{ij}-\frac{d_{ij}}{2\alpha }$, it can be expressed in vector form as:


(14) $$\underset{0\leq z_{ij}\leq 1,z_{i}^{⊤}1=1}{\min}\mathrm{||}z_{i}-c_{i}{\mathrm{||}}_{2}^{2}$$

By constructing the Lagrangian function, the optimal solution can be obtained using the Karush–Kuhn–Tucker (KKT) conditions (Stephen and Lieven 2004). The optimal solution can be expressed as: $z_{ij}={\left(g_{ij}-\frac{d_{ij}}{2\alpha }+\frac{\eta }{2}\right)}_{+}$. To satisfy $\sum_{j=1}^{n}z_{ij}=1$, ηis adjusted such that $\sum_{j=1}^{n}{\left(g_{ij}-\frac{d_{ij}}{2\alpha }+\frac{\eta }{2}\right)}_{+}=1$. By setting $\eta^{\prime }=\frac{\eta }{2}$, the final solution becomes:


(15) $$z_{ij}={\left(g_{ij}-\frac{d_{ij}}{2\alpha }+\eta^{\prime }\right)}_{+}$$

The entire optimization procedure of the proposed method is summarized in Algorithm 1.

Algorithm 1 Input: Multi-view data $X=\{X^{\left(v\right)}{\}}_{v=1}^{t}$, where each $X^{\left(v\right)}\in R^{d^{\left(v\right)}\times n}$ denotes the data matrix of the v-th omics view with $d^{\left(v\right)}$ features and n samples; parameters α and β; dimension of the latent representation d.

Output: Fused similarity graph Z

1. Initialization: Initialize W and H with random values. Compute A using formula (4).

2. while not converge do

3. Fixed H, Z to update W by Equation (10);

4. Fixed W, Z to update H by solving Equation (12);

5. Fixed H, W to update Z by Equation (15)

6. r = r + 1;

7. check convergence condition:

8. end while

**Figure 1:**
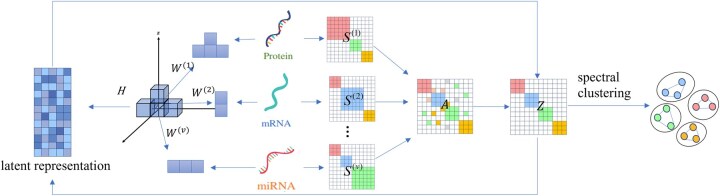
Schematic diagram of the ULSL method. W represents the projection matrix corresponding to each omics view; H represents the low-dimensional latent feature representation; $S^{\left(1\right)},S^{\left(2\right)},\ldots ,S^{\left(t\right)}$ are the similarity matrices for each omics view; A is the initial fused similarity matrix; Z represents the final fused similarity graph output after the algorithm converges.

After obtaining the fused similarity matrix *Z*, perform spectral clustering on get *Z* the clustering results. When there is no prior information about the number of clusters, use the eigengap method to determine the number of clusters. The proposed ULSL framework is illustrated in Fig. 1.

### 2.6 Computational complexity analysis

The computational complexity of solving Problem (6) consists of five components. First, updating each $W^{\left(v\right)}$using the ADMM algorithm has a complexity of $O(\sum_{v=1}^{t}\left(d^{\left(v\right)}nd+nd^{2}+d^{3}+d^{\left(v\right)}d^{2}\right))$, where $d^{\left(v\right)}$ is the dimensionality of the v-th view of the dataset and d is the dimension of the latent embedding representation. For updating H, the matrices E and Q are first computed, with computational complexities of $O(d^{2}\sum_{v=1}^{t}d^{\left(v\right)})$ and $O(dn\sum_{v=1}^{t}d^{\left(v\right)})$, respectively. Then, H is solved via the Sylvester equation, which has an approximate complexity of $O(n^{3})$. Therefore, the total complexity for updating H is roughly $O(d^{2}\sum_{v=1}^{t}d^{\left(v\right)}+dn\sum_{v=1}^{t}d^{\left(v\right)}+n^{3})$. Updating Z incurs a computational complexity of about $O(n^{2}d+n^{2}\log n)$.Consequently, for each iteration of the algorithm, the main computational complexity is approximately $O(dn\sum_{v=1}^{t}d^{\left(v\right)}+n^{3}+tn^{2}\log n)$.

## 3 Results

We designed a series of experiments to evaluate the effectiveness of the proposed algorithm, comparing it with nine representative methods. These include classic and advanced multi-omics clustering approaches such as SNF, CIMLR, NEMO, LRAcluster (Wu *et al.* 2015), iClusterBayes, MOSD, and MCLS. as well as state-of-the-art deep learning-based multi-view clustering frameworks, namely SCMVC and GDMVC. To ensure a rigorous and fair comparison regarding data usage, we uniformly evaluated all methods on the same set of high-dimensional multi-omics features (e.g. gene expression, DNA methylation, and miRNA expression) derived from overlapping patient cohorts. For parameter settings, we followed the implementation details and parameter choices reported by Rappoport and Shamir (2018) for SNF, NEMO, LRAcluster, and iClusterBayes, while adhering strictly to the original guidelines for CIMLR (Ramazzotti *et al.* 2018), MOSD (Duan *et al.* 2024), MCLS (Ye *et al.* 2023), SCMVC (Wu *et al.* 2024), and GDMVC (Bai *et al.* 2024). further details are provided in Supplementary Note 1. The proposed ULSL method involves three hyperparameters, denoted as α, β, and d: α was determined by the ratio between the number of features and the number of samples, with $\alpha =\alpha_{0}\sum_{v=1}^{t}d^{\left(v\right)}/n$, $\alpha_{0}$ within the range [2, 3], β was fixed at 0.1, and d was explored within the range [20, 120]. The optimal parameter combination is selected using a grid search strategy. We conducted simulation studies and real-data experiments to evaluate the ability to capture global clustering structures and their practical utility in clinical applications.

### 3.1 Simulation study

The simulation datasets were generated following the design proposed by Shi *et al.* (2017). Specifically, we simulated three types of omics data across four patient subtypes, resulting in a total of 400 samples. These samples were constructed from real genomic atlas data using singular value decomposition (SVD) under a predefined clustering structure. Each individual omics dataset was designed to distinguish only three of the subtypes, while the integration of all three datasets allowed for the separation of all four subtypes (Fig. S1). The data were derived from GSE51557 (Conway *et al.* 2015), GSE73002 (Shimomura *et al.* 2016), and GSE10645 (Nakagawa *et al.* 2008), corresponding to DNA methylation, miRNA expression, and RNA expression, respectively. Based on these, we constructed two datasets (SimData1 and SimData2), where SimData1 exhibits clear boundaries between subtypes, while SimData2 presents more ambiguous boundaries. We evaluated all algorithms under different noise levels (low and high) and varying signal strengths (represented by the proportions of differentially expressed features at 5% and 10%). Specific implementation details are provided in Supplementary Note 2. For each condition, we conducted 200 independent runs to mitigate random variation. Normalized Mutual Information (NMI) was used as the primary performance metric, and results were averaged over the 100 repetitions for each noise and signal condition.

The results are shown in Table 1. When the subtype boundaries are clear (SimData1), the performance of ULSL is comparable to that of other methods. Under varying signal and noise conditions, all methods can stably and accurately identify the four subtypes. However, when the subtype boundaries are blurred (SimData2), the performance of all methods decreases to varying degrees. SNF can still achieve high accuracy under low noise levels, but its performance rapidly declines with increasing noise or varying proportions of signal features. In contrast, ULSL consistently outperforms other methods across all signal and noise conditions. Even under the most extreme conditions set in this experiment (i.e. high noise and low signal levels), the NMI of the ULSL method still reaches 0.41, which is considerably higher than that of the second-best method, iClusterBayes (0.33). Overall, ULSL demonstrates significantly better data adaptability than SNF and other comparative methods, showing stable and superior global sample recognition under all conditions.

**Table 1:** Results of applying the 10 algorithms on simulation datasets.

Data	Noise	Signal	SNF	CIMLR	NEMO	LRAcluster	iClusterBayes	MOSD	CLMS	SCMVC	GDMVC	ULSL
SimData1	Low	5%	0.99 ± 0.01	0.78 ± 0.07	1.00 ± 0.00	0.72 ± 0.09	0.87 ± 0.16	1.00 ± 0.03	0.63 ± 0.09	0.99 ± 0.05	1.00 ± 0.00	1.00 ± 0.00
		10%	0.99 ± 0.01	0.77 ± 0.06	1.00 ± 0.00	0.71 ± 0.10	0.88 ± 0.15	1.00 ± 0.00	0.65 ± 0.07	0.99 ± 0.04	1.00 ± 0.00	1.00 ± 0.00
	High	5%	0.99 ± 0.01	0.77 ± 0.06	1.00 ± 0.00	0.72 ± 0.09	0.86 ± 0.17	1.00 ± 0.00	0.55 ± 0.14	0.98 ± 0.06	1.00 ± 0.00	1.00 ± 0.00
		10%	0.99 ± 0.01	0.77 ± 0.06	1.00 ± 0.00	0.72 ± 0.09	0.86 ± 0.17	1.00 ± 0.00	0.62 ± 0.10	0.99 ± 0.05	1.00 ± 0.00	1.00 ± 0.00
SimData2	Low	5%	0.51 ± 0.10	0.33 ± 0.08	0.58 ± 0.11	0.23 ± 0.08	0.51 ± 0.13	0.52 ± 0.09	0.13 ± 0.06	0.60 ± 0.14	0.58 ± 0.31	0.72 ± 0.14
		10%	0.73 ± 0.10	0.46 ± 0.08	0.82 ± 0.09	0.37 ± 0.08	0.66 ± 0.16	0.74 ± 0.12	0.20 ± 0.08	0.78 ± 0.12	0.76 ± 0.23	0.91 ± 0.08
	High	5%	0.25 ± 0.09	0.18 ± 0.08	0.30 ± 0.09	0.11 ± 0.06	0.33 ± 0.10	0.29 ± 0.10	0.06 ± 0.03	0.38 ± 0.15	0.35 ± 0.12	0.41 ± 0.14
		10%	0.49 ± 0.10	0.32 ± 0.08	0.58 ± 0.11	0.23 ± 0.08	0.51 ± 0.15	0.51 ± 0.09	0.13 ± 0.05	0.60 ± 0.15	0.66 ± 0.19	0.71 ± 0.14

Note: Values represent the mean ± standard deviation of NMI across 200 synthetic datasets under varying conditions. Signal indicates the proportion of differentially expressed features.

### 3.2 Real data study

In this study, we utilized multi-omics data from TCGA to evaluate the performance of various multi-view clustering methods in identifying cancer subtypes. Specifically, we adopted the datasets provided by Rappoport and Shamir (2018), which include 10 cancer types: acute myeloid leukemia (AML), breast invasive carcinoma (BIC), colon adenocarcinoma (COAD), glioblastoma multiforme (GBM), kidney renal clear cell carcinoma (KIRC), liver hepatocellular carcinoma (LIHC), lung squamous cell carcinoma (LUSC), ovarian serous cystadenocarcinoma (OV), sarcoma (SARC), and skin cutaneous melanoma (SKCM). For each cancer type, three omics data modalities were available: mRNA expression, DNA methylation, and miRNA expression. All TCGA datasets are publicly accessible at http://acgt.cs.tau.ac.il/multiomic_benchmark/download.html.

We conducted preprocessing on the obtained TCGA data. For all cancer types, missing values in the clinical data were removed, and the intersection of samples across different omics datasets was taken to determine the number of patients. Both mRNA and miRNA data were log-transformed, and for each omics modality (mRNA, DNA methylation, and miRNA), features with zero variance were excluded. Further details of these datasets are summarized in Table 2.

**Table 2:** Overview of 10 TCGA data sets.

Cancer	Patients (*n*)	mRNA (No. of features)	Methylation (No. of features)	miRNA (No. of features)
AML	159	19 900	5000	557
BIC	621	20 221	5000	885
COAD	213	19 988	5000	612
GBM	271	12 042	5000	534
KIRC	181	20 118	5000	797
LIHC	361	20 146	5000	849
LUSC	337	20 235	5000	880
OV	286	20 176	5000	616
SARC	258	20 221	5000	838
SKCM	437	20 225	5000	900

Note: The “Patients” column shows the number of samples (*n*) used in the study. The numbers in the “mRNA,” “Methylation,” and “miRNA” columns represent the total count of molecular features (e.g. genes, CpG sites, and miRNAs) retained for each omics type after data preprocessing and feature selection.

We evaluated the effectiveness of cancer subtype identification using two complementary metrics: survival analysis and clinical enrichment analysis. For survival analysis, the Cox log-rank test was employed to assess differences in survival outcomes among subtypes, where smaller *P*-values indicate more significant differences in survival profiles. For clinical enrichment analysis, six clinical variables were considered: age at initial diagnosis, gender, pathologic T, pathologic N, pathologic M, and pathologic stage. Not all cancer types contain all of the aforementioned clinical labels. The specific clinical labels available for each cancer type are listed in Table S1. Enrichment for categorical variables was assessed using the χ^2^ test of independence, while enrichment for continuous variables was evaluated using the Kruskal–Wallis test. Following the approach described by Rappoport and Shamir (2018), permutation tests were used to assess the statistical significance of clinical variables. The detailed implementation of the permutation test is provided in the Supplementary Note 3. Briefly, empirical *P*-values were estimated by randomly permuting cluster labels across samples and computing the corresponding test statistics; all *P*-values reported in this study were obtained using this procedure. After completing the permutation tests, multiple hypothesis correction was applied to account for the testing of multiple clinical variables. Specifically, for each cancer type and each method, Bonferroni correction was performed at a significance level of 0.05.

The number of clusters is determined with reference to the method proposed by Yang *et al.* (2021) in their paper, and the search range [k_1_, k_2_] was set according to the sample size of each dataset as follows: when the sample size was greater than 500, k_1_ = 4 and k_2_ = 8; when the sample size was between 200 and 500, k_1_ = 3 and k_2_ = 6; and when the sample size was less than 200, k_1_ = 2 and k_2_ = 5. The optimal number of clusters was then automatically determined using the gap statistic method, and the corresponding clustering results were directly obtained.

**Figure 2:**
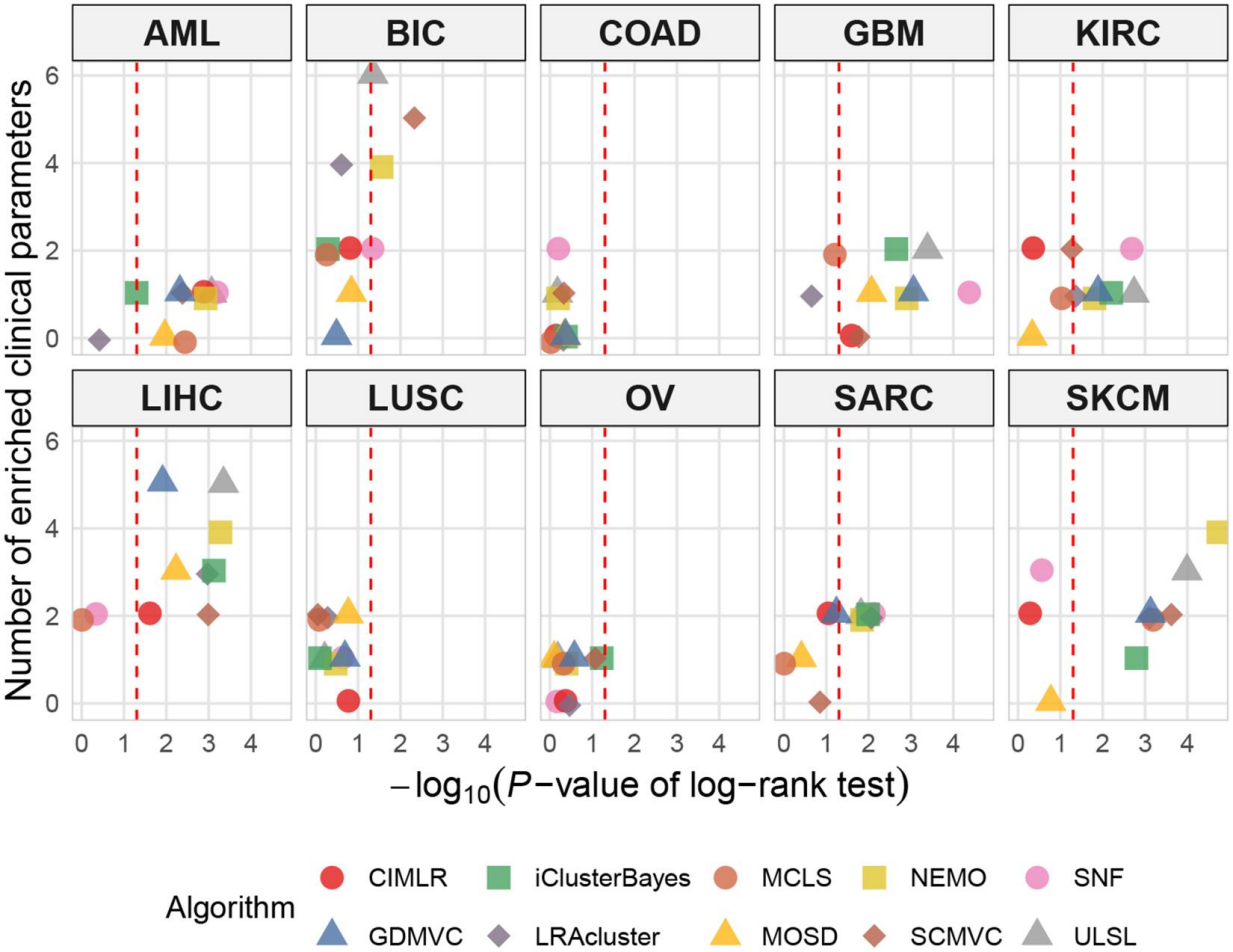
Results of 10 clustering algorithms across 10 cancer types. In each plot, the X-axis represents −log_10_  *P*-value from the log-rank test, and the Y-axis indicates the number of clinical parameters enriched within the clusters. The red vertical line denotes the threshold for significant survival differences (*P* < 0.05).

**Figure 3:**
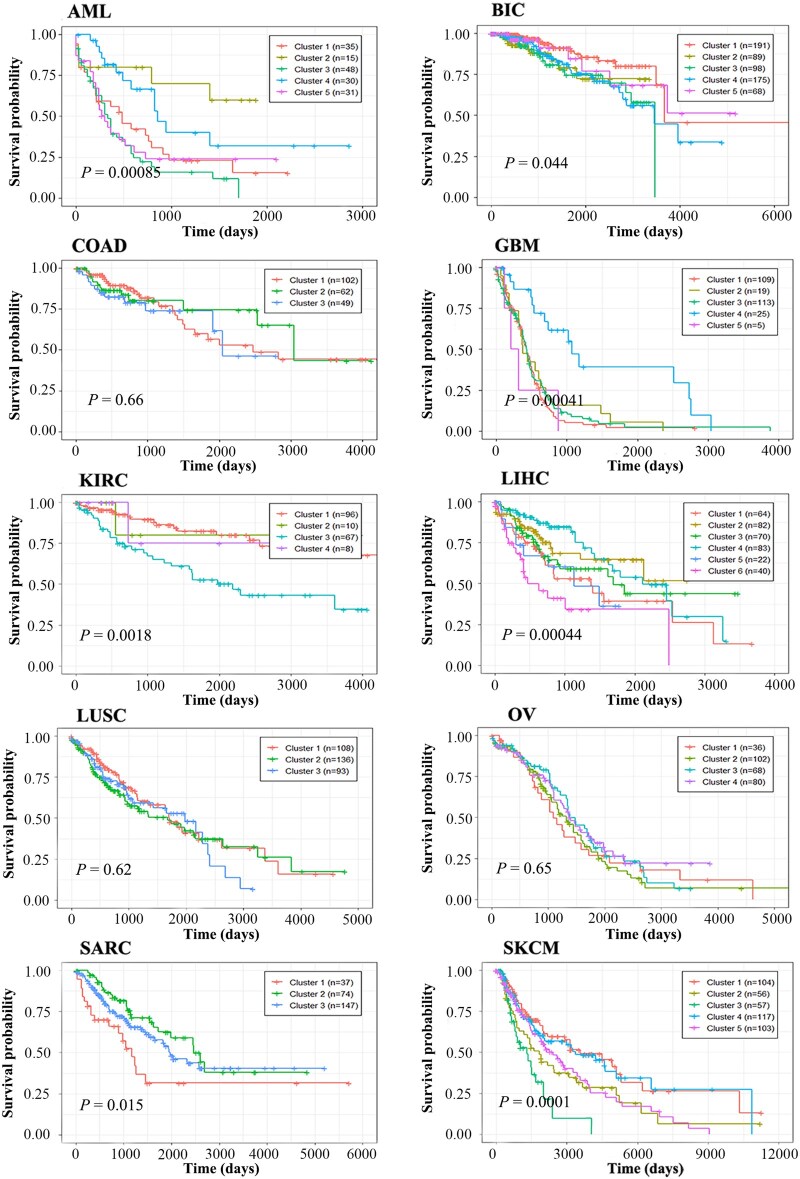
Kaplan-Meier survival analysis of 10 TCGA cancer cohorts based on molecular subtypes identified by the ULSL algorithm.

Table 3 and Fig. 2 summarize the performance of the 10 algorithms across 10 cancer datasets. In survival analysis, the ULSL method identified survival-associated clusters in 7 out of 10 cancer types, outperforming all other methods (the maximum for any other method was 6). And no method was able to detect significant survival differences in the COAD and LUSC datasets. Figure 3 illustrates the Kaplan–Meier survival curves of the cancer subtypes identified by ULSL across the 10 cancer types.

**Table 3:** Comparison of survival analysis and clinical enrichment analysis results of 10 clustering algorithms on the TCGA dataset.

Cancer/Alg	SNF	CIMLR	NEMO	LRAcluster	iClusterBayes	MOSD	MCLS	SCMVC	GDMVC	ULSL
AML	1/**3.19**	0/0.74	1/**2.92**	0/0.39	1/**1.30**	0/**2.01**	0/**2.44**	1/**2.38**	1/**2.32**	1/**3.07**
BIC	2/**1.34**	2/0.81	4/**1.55**	4/0.61	2/0.29	1/0.84	2/0.26	5/**2.33**	0/0.49	6/**1.35**
COAD	2/0.20	0/0.16	0/0.19	0/0.32	0/0.38	0/0.33	0/0.03	1/0.33	0/0.37	1/0.18
GBM	1/**4.38**	0/**1.60**	1/**2.90**	1/0.65	2/**2.65**	1/**2.07**	2/1.20	0/**1.77**	1/**3.06**	2/**3.39**
KIRC	2/**2.69**	2/0.36	1/1.21	1/**1.36**	1/**2.20**	0/0.33	1/1.03	2/1.27	1/**1.89**	1/**2.74**
LIHC	2/0.34	2/**1.61**	4/**3.28**	3/**2.97**	3/**3.12**	3/**2.23**	2/0.01	2/**2.99**	5/**1.91**	5/**3.35**
LUSC	1/0.64	0/0.77	1/0.47	2/0.28	1/0.10	2/0.77	2/0.08	2/0.05	1/0.69	1/0.21
OV	0/0.17	0/0.37	1/0.39	0/0.46	1/1.22	1/0.10	1/0.32	1/1.07	1/0.58	1/0.19
SARC	2/**2.12**	2/1.05	2/**1.84**	2/**2.06**	2/**1.98**	1/0.41	1/0.01	0/0.85	2/1.24	2/**1.82**
SKCM	3/0.56	2/0.29	4/**3.73**	2/**3.10**	1/**2.80**	0/0.77	2/**3.19**	2/**3.62**	2/**3.13**	3/**3.99**
#sig	16/5	10/2	20/6	15/4	14/6	9/3	13/2	16/5	14/5	**23/7**

Note: In each cell A/B, A is the number of enriched clinical parameters, B is the −log10 *P*-value of survival analysis #sig is all the number of datasets with significant clinical/survival results. The bold values are significant results and we use 0.05 as the threshold for significant.

In clinical enrichment analysis, the ULSL method detected at least one significant clinical factor in 9 out of 10 datasets, the same as methods NEMO, SNF, and iClusterBayes, thereby achieving the largest number. However, in terms of the total number of significant clinical factors identified, ULSL achieved the best result with 23 significant clinical labels, higher than the second-best method, which identified 20. As shown in Fig. 2, ULSL was able to identify a greater number of significant clinical labels in the majority of cancer types. The results of clinical parameter enrichment for each method are provided in the Supplementary Materials 2. The optimal number of clusters obtained by each method is listed in Table S2.

Since the performance of clustering methods should be evaluated based on both survival analysis and clinical enrichment analysis, the results collectively demonstrate that ULSL consistently achieved the highest number of survival-associated clusters and the largest number of significant clinical labels across the 10 cancer types. These findings highlight the superior competitiveness of ULSL in cancer subtype identification on the TCGA datasets.

### 3.3 Parameter sensitivity and convergence analysis

We evaluated the robustness of ULSL with respect to the parameter *α* and the latent dimension d. We first performed sensitivity analyses on the previously described multi-omics simulated datasets. Specifically, we selected the SimData2 dataset and evaluated it under various combinations of noise levels and signal strengths. NMI was used as the performance metric, and each experiment was repeated 100 times with different random seeds, with the average result reported. Subsequently, we performed the same evaluation on 10 TCGA datasets, where the −log10 *P*-value from the Cox log-rank test was employed to assess clinical relevance.

**Figure 4:**
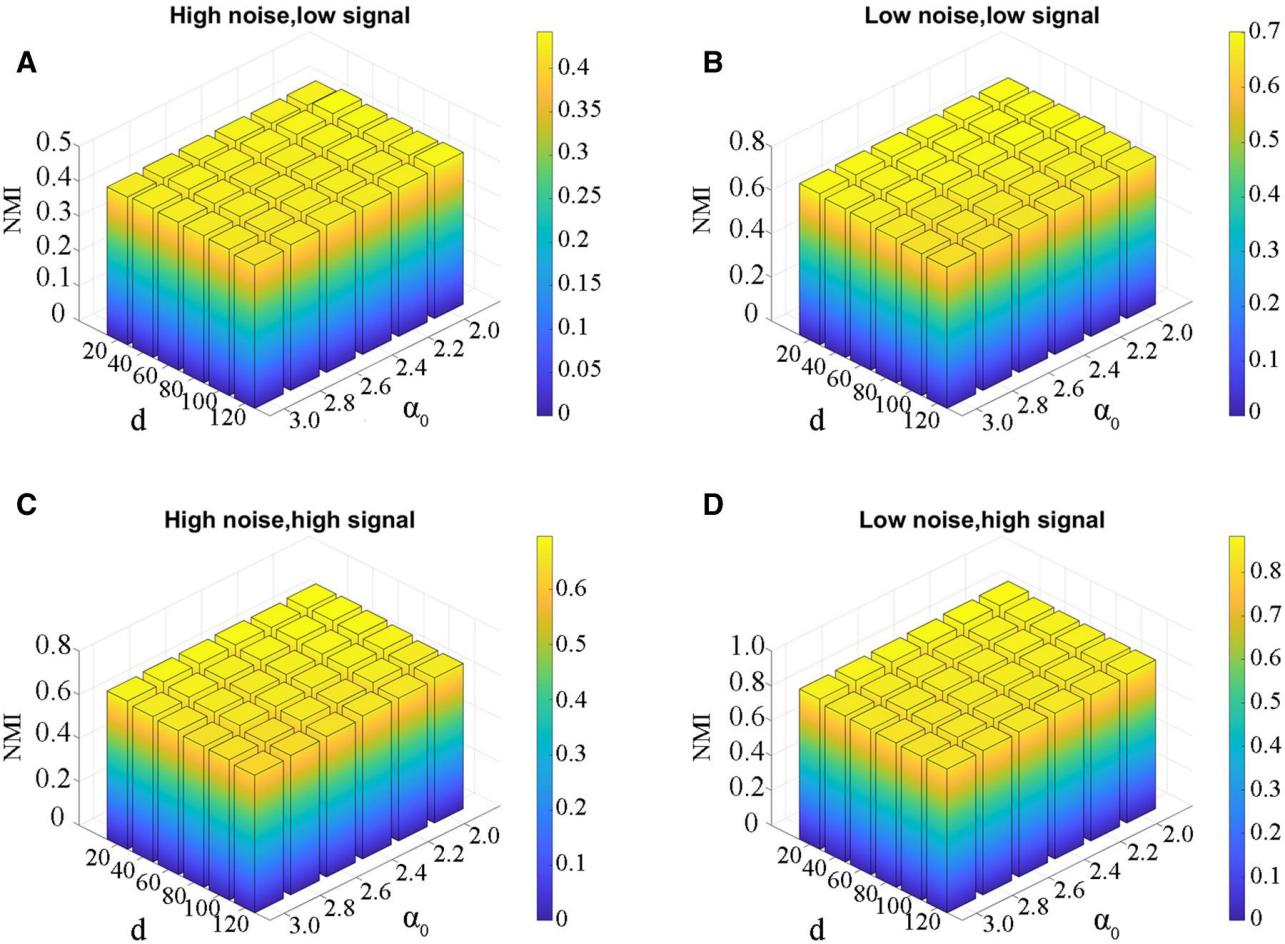
The impact of different parameter settings on the clustering performance (NMI) of the ULSL method under varying noise and signal levels. (A) High noise, low signal; (B) Low noise, low signal; (C) High noise, high signal; (D) Low noise, high signal.

**Figure 5:**
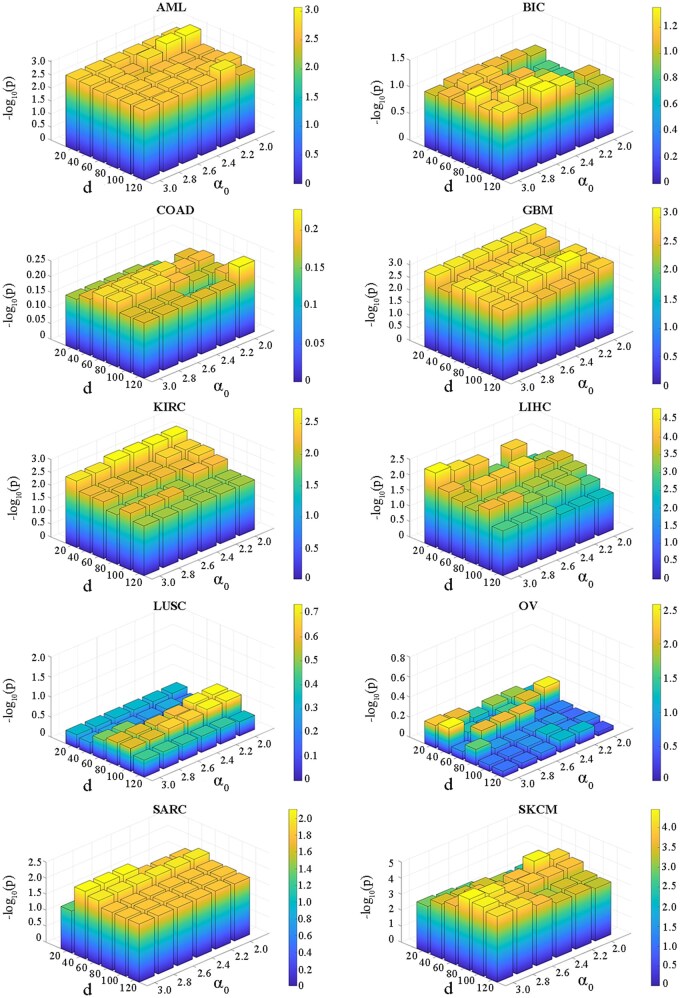
The results show the variations in −log10 *P*-value scores (derived from the Cox log-rank test) across different hyperparameter configurations of the ULSL algorithm on TCGA datasets. The stability of the scores indicates the robustness of ULSL in identifying clinically meaningful cancer subtypes.

For both parameters *α* and *d*, we applied a grid search within their predefined ranges to identify the optimal values. All results generated during the grid search were systematically recorded and are presented in Figs. 4 and 5. From these figures, we can observe that the two evaluation metrics of the proposed method remain relatively stable within the search range, indicating that ULSL is robust to the loss function parameters.

In addition, we analyzed the convergence of the proposed method. Figure S2 illustrates the objective function values over iterations on the TCGA datasets. It can be observed that the proposed method exhibits good convergence, as the loss function consistently decreases with the number of iterations and eventually stabilizes across all datasets.

## 4 Case study: TCGA acute myeloid leukemia (AML)

**Figure 6:**
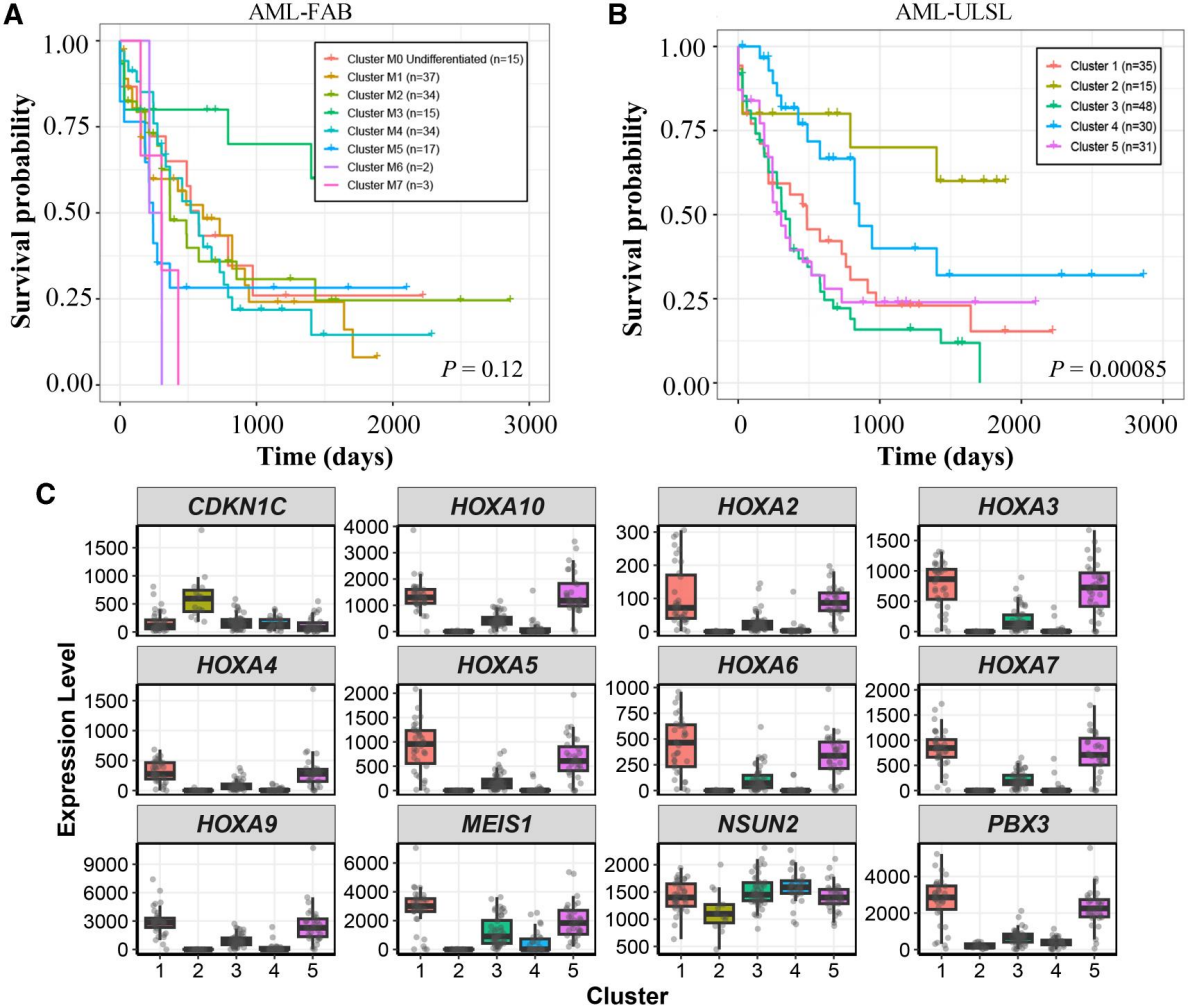
(A) Kaplan-Meier survival curves of patients stratified by FAB subtypes (Log-rank test,P = 0.12). (B) Kaplan-Meier survival curves of patients stratified by ULSL clustering. ULSL clustering showed significant survival differences (Log-rank test,$P=8.5\times 10^{-4}$). (C) Box plots showing the expression levels of key differential genes across AML subtypes defined by ULSL clustering.

We systematically compared the prognostic value of ULSL-derived clustering with the conventional French–American–British (FAB) morphological classification. FAB classification is a well-established clinical system for acute myeloid leukemia (AML) (Bennett *et al.* 1976), primarily based on the quantitative composition and morphological characteristics of hematopoietic cells in peripheral blood and bone marrow. Using FAB subtypes and ULSL clusters as grouping variables, we performed log-rank survival analyses, yielding *P*-values of 0.12 (Fig. 6A) and 8.5 × 10^−4^ (Fig. 6B), respectively. These results demonstrate that ULSL clustering provides a markedly superior stratification of patient survival, highlighting its strong prognostic discrimination capability.

To further characterize the clinical and biological features underlying each ULSL cluster, we conducted enrichment analyses of clinical annotations and FAB subtypes. Distinct clusters exhibited pronounced differences across multiple dimensions, including FAB distribution, cytogenetic risk categories, age profiles, and hematological parameters. Table 4 presents how FAB morphological classifications and CALGB cytogenetic risk groups are distributed among the different AML clusters. Comprehensive clustering results and enriched clinical annotations are provided in the Supplementary Materials 3.

**Table 4:** Distribution of FAB morphological codes and CALGB cytogenetic risk categories across five AML clusters.

Subtypes identified by ULSL	FAB morphology code								CALGB cytogenetics risk category		
	M0	M1	M2	M3	M4	M5	M6	M7	Favorable	Normal	Poor
Cluster 1	3	17	9	0	5	1	0	0	0	29	4
Cluster 2	0	0	0	15	0	0	0	0	14	1	0
Cluster 3	12	10	12	0	8	0	2	3	1	24	23
Cluster 4	0	8	12	0	9	0	0	0	16	11	3
Cluster 5	0	2	1	0	12	16	0	0	0	29	2

Note: The numerical entries represent the count of patients. This table tabulates the distribution of patients across different ULSL clusters, stratified by their FAB morphological subtypes and CALGB cytogenetic risk categories.

Notably, FAB subtypes M1, M2, and M4 were predominantly distributed across two short-survival clusters, cluster 1 and cluster 3, yet these two clusters displayed substantially different clinical characteristics. Cluster 1 was significantly enriched for patients without known recurrent genetic abnormalities and was characterized by elevated white blood cell counts and reduced bone marrow lymphocyte percentages. In contrast, cluster 3 showed an opposing hematological profile and was associated with markedly adverse prognostic features. Patients in this cluster were generally older and exhibited a strong enrichment for the FAB M0 (Undifferentiated) subtype. Among the 15 patients classified as M0, 12 were assigned to cluster 3. Consistent with prior evidence linking M0 AML to poor outcomes (Béné *et al.* 2001), this cluster also concentrated nearly all patients with FAB M0, M6, and M7 subtypes, as well as those categorized as high-risk according to the CALGB cytogenetic classification, collectively defining a prototypical high-risk AML phenotype. In contrast, cluster 4 demonstrated a distinctly different clinical profile, characterized by a younger patient population and relatively favorable survival outcomes, suggesting that it may represent a biologically less aggressive AML subgroup. Cluster 5 was strongly enriched for the FAB M5 subtype, corresponding to acute monocytic leukemia. Patients in this cluster were almost exclusively classified as intermediate cytogenetic risk by CALGB criteria and exhibited markedly elevated monocyte counts, in close agreement with the canonical hematological features of M5 AML. Cluster 2 displayed an exceptionally homogeneous and distinctive clinical pattern. All 15 patients within this cluster were classified as FAB M3, and conversely, all M3 cases in the cohort were exclusively assigned to this cluster. Furthermore, these patients were predominantly categorized within the favorable CALGB cytogenetic risk group. Importantly, FAB M3 corresponds to acute promyelocytic leukemia (APL) which widely recognized for its favorable prognosis (Wang and Chen 2008). Consistent with this established biology, cluster 2 exhibited significantly superior survival outcomes compared with all other clusters.

Subsequently, we investigated the biological relevance of each subtype at the molecular level. Statistically, we first employed the Kruskal–Wallis H test to screen for global differential features across multi-omics data. Following this, the Wilcoxon rank-sum test was applied in a “one-versus-rest” manner to identify subtype-specific biomarkers. All resulting *P*-values were adjusted using the Benjamini–Hochberg method to control the False Discovery Rate (FDR). Functional enrichment analyses, including GO and KEGG (as shown in Figs. S3–S7), further elucidated the molecular mechanisms underlying the observed survival disparities.

Prognostic variations among different acute myeloid leukemia (AML) subtypes are rooted in distinct molecular foundations (Fig. 6C). The high-risk subtypes with the poorest survival (Clusters 1, 3, and 5) generally exhibit high expression of the HOXA family and PBX3, suggesting highly active stemness signaling, enhanced invasiveness, and prominent immune evasion (Li *et al.* 2013; Chen *et al.* 2019). In contrast, the favorable-prognosis Subtype 2 demonstrates a unique molecular protective mechanism: it not only shows inhibition of stemness pathways such as the HOXA family, reducing the self-renewal capacity of AML cells, but also displays an NSUN2-low/CDKN1C-high expression pattern that is diametrically opposed to that of high-risk subtypes. As a CDK inhibitor of the Cip/Kip family, CDKN1C is extensively involved in cell cycle control, differentiation, and apoptosis, yet it is frequently downregulated in various human cancers (Ru *et al.* 2017; Qiu *et al.* 2018). Previous studies have confirmed that NSUN2 expression is negatively correlated with CDKN1C, exerting oncogenic effects by repressing CDKN1C (Mei *et al.* 2020). Consequently, the NSUN2-low/CDKN1C-high signature in Subtype 2 relieves the repression of this tumor suppressor and activates cell cycle arrest, effectively inhibiting leukemia cell proliferation and forming the molecular basis for its favorable prognosis. Additionally, the differential genes in Subtype 4 are involved in metabolism and extracellular matrix (ECM) remodeling, consistent with its relatively lower invasiveness.

In summary, ULSL clustering not only outperforms the traditional FAB classification in prognostic stratification, but also reveals substantial clinical and biological heterogeneity within FAB-defined subtypes. These findings underscore the potential of ULSL as a powerful framework for refined AML subtyping and risk stratification beyond conventional morphology-based systems.

## 5 Discussion

In this study, we propose a novel multi-cancer subtype identification method named ULSL. This approach automatically learns low-dimensional latent representations from all omics data, capturing the inherent high-order correlations and complementary information within multi-omics data. The integrated fused similarity matrix not only consolidates the structural characteristics of multi-omics data at a global level but also reciprocally refines the learning of latent representations. This process enhances strong similarities between samples while reducing spurious associations and mitigating noise. Finally, by performing spectral clustering on the consensus similarity matrix, more stable and biologically meaningful cancer subtype classifications are obtained.

Extensive experiments on both synthetic data and 10 TCGA cancer datasets, including survival analysis and clinical enrichment analysis, demonstrate that the proposed ULSL method achieves superior clustering performance compared with seven state-of-the-art multi-omics clustering approaches. In simulation studies, ULSL effectively suppresses noise from raw data and shows strong robustness in handling weak-signal features, leading to more reliable clustering outcomes. Moreover, empirical evaluation on the TCGA datasets further highlights its advantages over existing methods: (i) ULSL identifies survival-associated subtypes in a larger number of cancer types, revealing potential differences in clinical prognosis; and (ii) ULSL achieves a higher number of significantly enriched clinical parameters, indicating its ability to better capture associations between molecular features and clinical characteristics. Furthermore, the concordance between ULSL-identified AML subtypes and established cancer subtype studies supports the biological relevance of the subtypes uncovered by our approach.

From a clinical perspective, these findings are of particular importance. The proposed method not only enables more accurate identification of cancer subtypes and improved patient stratification but also provides potential molecular evidence to guide personalized treatment and clinical decision-making. For example, the ability to detect subtypes associated with survival differences may help oncologists design more targeted therapeutic strategies, while significantly enriched clinical parameters could serve as valuable biomarkers for diagnosis and prognosis prediction (Ferro *et al.* 2025; Gao *et al.* 2025; Hong *et al.* 2025; Wang *et al.* 2025).

This study still has several limitations. First, our current algorithm can only be applied to datasets with complete multi-omics profiles, which substantially restricts the number of usable samples. Second, the current study lacks validation in an independent cohort. While external validation is crucial for assessing generalizability, matched multi-omics datasets (specifically mRNA, DNA methylation, and miRNA) for AML are extremely scarce in public repositories. Validating our findings with only a single omics layer would be methodologically inconsistent with our multi-view framework and may result in non-rigorous conclusions. Moreover, although the method achieves superior performance in cancer subtype identification, it is less efficient in terms of runtime. The iterative updates between latent representation learning and similarity matrix construction increase the overall computational complexity, making it less advantageous compared with some non-iterative multi-omics clustering approaches. In future work, we plan to extend the method to handle datasets with missing omics and to improve its computational efficiency.

## Supplementary Material

vbag110_Supplementary_Data

## Data Availability

The data underlying this article are available in The Cancer Genome Atlas (TCGA) repository, which can be accessed via the Genomic Data Commons (GDC) Data Portal at https://portal.gdc.cancer.gov/.
