# Supplementary material for: ULSL: Unified Latent and Similarity Learning for robust multi-omics cancer subtype identification
Source: Bioinform Adv. 2026 Apr 17;6(1):vbag110. doi: 10.1093/bioadv/vbag110 (PMC13138254; doi:10.1093/bioadv/vbag110)
Supplement: vbag110_Supplementary_Data [file vbag110_supplementary_data.zip › Supplementary Material.docx]

**Supplementary Note 1: Preprocessing, execution details, and implementation of the comparison methods.**

**Preprocessing:**

All features were then normalized to have zero mean and standard deviation 1.

**SNF**

**Execution details:**

The affinity matrices for each omics dataset were computed using the dist2 and affinityMatrix functions from the SNFtool package, with the number of neighbors set to one-tenth of the total sample size and sigma = 0.5. These matrices were then integrated using the SNF method, employing the same number of neighbors and iterating 30 times. Spectral clustering was subsequently applied to the fused matrix using default parameters.The optimal number of clusters was chosen using the rotation method [1] on the integrated matrix.

**Implementation:**

The functions dist2, affinityMatrix, SNF, and spectralClustering from the SNFtool package (version 2.3.0) were employed in the analysis.

References:

[1] Zelnik-Manor, L. and Perona, P. (2004). Self-tuning spectral clustering. In Proceedings of the 17th International Conference on Neural Information Processing Systems, pages 1601–1608. MIT Press.

**CIMLR**

**Execution details:**

Following the procedure described in the original paper, we first applied the functions CIMLR_Estimate_Number_of_Clusters and NUMC to determine the optimal number of clusters within the range of 2 to 10. Based on the estimated cluster number, we then performed clustering using the CIMLR function.We set the number of clusters c to the optimal value obtained from the NUMC function. In the CIMLR function, the parameter cores.ratio was set to 0 to disable parallel computing.

**Implementation:**

We employed the CIMLR function from the CIMLR package (version 1.0.0), which can be downloaded from https://github.com/danro9685/CIMLR.

**NEMO**

**Execution details:**

We used the nemo.affinity function to compute the integrated similarity matrix across multiple omics datasets. The number of neighbors for each omic was set to one-sixth of the sample size. Spectral clustering was then applied to the integrated matrix with default parameters.We applied the rotation method [1] to the integrated similarity matrix in order to estimate the optimal number of clusters within the range of 2 to 10.

**Implementation:**

We implemented the method using the NEMO R package provided by the authors, which can be downloaded from <https://github.com/Shamir-Lab/NEMO>.

**LRAcluster**

**Execution details:**

LRAcluster was applied to the data assuming a Gaussian data type with default parameters. Clustering was performed on the low-dimensional representation using K-means with default settings. To determine the latent dimension, LRAcluster was run with dimensionality ranging from 1 to 10, and the optimal dimension was selected using the elbow method. To determine the number of clusters, we applied K-means clustering on the low-dimensional data with k=2–10, each with 60 random initializations, and selected the solution with the lowest silhouette score.

**Implementation:**

LRAcluster was implemented using the LRAcluster R package (version 1.0, available at <http://bioinfo.au.tsinghua.edu.cn/member/jgu/lracluster/>), while K-means clustering was performed using the built-in kmeans function in R.

**iClusterBayes**

**Execution details:**

iClusterBayes was applied to the data using the Gaussian data type with default parameters. The dimensionality was determined according to the deviance ratio criterion defined by the original authors, selecting the dimension corresponding to the maximum deviance ratio. The number of clusters was then set to “dimension + 1”.

**Implementation:**

We employed the tune.iClusterBayes function from the iClusterPlus R package (version 1.45.0). iClusterBayes was run in parallel across all low-dimensional spaces ranging from 1 to 9 dimensions.

**MOSD**

**Execution details:**

The clustering number is estimated by seperarion cost method.

**Implementation:**

We implemented the method using the MOSD function from the MOSD package (version 0.1.0),which can be downloaded from https://github.com/DXCODEE/MOSD.

**MCLS**

**Execution details:**

In the experiments, all parameters were set to their default values, and the complete dataset was used for analysis. The multi-omics data were integrated via the data_col parameter to obtain the latent embedding. Subsequently, spectral clustering was applied in the latent subspace using the specClust function, where the number of clusters was automatically determined by the algorithm.

**Implementation:**

The code was installed and used from <https://github.com/ShangCS/MCLS>.

**SCMVC**

**Execution details:**

SCMVC was applied to the data following the parameter settings specified in the training script. The deep neural network was optimized using the Adam optimizer with a learning rate of . The dimensionality of the view-specific features and the high-level consensus features were set to 64 and 20, respectively. The temperature parameter () in the contrastive loss function was fixed at 1. The training process involved 200 epochs of pre-training (reconstruction objective only) followed by 50 epochs of contrastive fine-tuning (joint reconstruction and consistency objectives). To determine the optimal number of clusters, we applied K-means clustering with ranging from 2 to 10, and selected the solution with the highest silhouette score.

**Implementation**

SCMVC was implemented using Python (version 3.11.5) and the PyTorch framework (version 2.1.0). The K-means clustering and silhouette score calculations were performed using the scikit-learn library (version 1.8.0). The code was installed and used from https://github.com/SongwuJob/SCMVC

**GDMVC**

**Execution details:**

GDMVC was applied to the multi-omics data with hyperparameters strictly adhering to the default settings recommended in the original study. Specifically, the model utilized a deep adaptive graph autoencoder architecture with hidden layer dimensions set to [256, 64]. The model was trained using the Adam optimizer with a learning rate of . The training process consisted of two stages: a pre-training phase of 10 epochs (with 100 iterations per epoch) to initialize the graph structure, and a fine-tuning phase of 100 epochs to optimize the consensus representation. For the adaptive graph construction, the number of neighbors was initialized to 6 and incremented by 5 during the graph update process. Clustering was performed on the final fused low-dimensional representations using K-means with default settings. To determine the optimal number of clusters, we applied K-means clustering with ranging from 2 to 10, and selected the solution with the highest silhouette score.

**Implementation**

GDMVC was implemented using Python (version 3.11.5) and the PyTorch framework (version 2.1.0). The K-means clustering and silhouette score calculations were performed using the scikit-learn library (version 1.8.0). The code was installed and used from https://github.com/yff-java/GDMVC/#

**Supplementary Note 2 Synthetic Data for Simulation Study**

The synthetic datasets for the simulation study were generated following the procedure described by Meng et al. (2016). First, three real biological datasets were prepared: GSE51557 (Conway et al., 2015), GSE73002 (Shimomura et al., 2016), and GSE10645 (Nakagawa et al., 2008), corresponding to different data types or layers, namely DNA methylation, miRNA expression, and RNA expression. From each dataset, 400 samples were randomly selected to construct the respective data matrices, denoted as X1, X2​, and X3, where rows represent features and columns represent samples. Each of these data matrices (X1​, X2​, and X3​) was then subjected to singular value decomposition (SVD), expressed as .

, ,

To preserve all biological features of the data, only the V matrices were modified to construct a clear four-class predefined clustering structure (Meng et al., 2015). Accordingly, three new data matrices with the same dimensions as X1, X2​, and X3​ were generated using the following formulation:

Here, represents the random noise of the expression value of feature i in sample j, and represents the average expression level for the signal features in each cluster.

In both SimData1 and SimData2, no single data type was sufficient to fully distinguish all four clusters. Specifically, each data type revealed only three observable subgroups: for example, in ​, samples 1–100, 101–300, and 301–400 formed three clusters; in ​, clusters comprised samples 1–100/201–300, 101–200, and 301–400; and in ​, clusters were formed by samples 1–200, 201–300, and 301–400. For each cluster in the matrices ,and ​, the cluster-specific mean values were randomly sampled without replacement from the set {0,2,4,6}, ensuring that the integrated data matrices preserved the underlying true structure.

To evaluate the performance of ULSL under different noise intensities (including low and high noise levels), we set different standard deviations σ for the normal distribution . For within-data-type noise levels, we set σ=1 for each data type to represent low noise levels and σ=2 to represent high noise levels.

Furthermore, considering that in real omics data only a small fraction of genes exhibit differential expression, we introduced only a limited number of signal features: 5% or 10% of all features were designated as differentially expressed. The procedure for generating SimData2 was largely the same, except that the signal strength was weakened; that is, the differences in between clusters were reduced and randomly sampled from {0,0.25,0.5,0.75}.

Next, the SVD of ,and ​ was computed to generate orthogonal matrices *V* (, , ) with clearly defined cluster structures:

, ,

Finally, the simulated datasets , , ​ were generated using the following formulation:

, ,


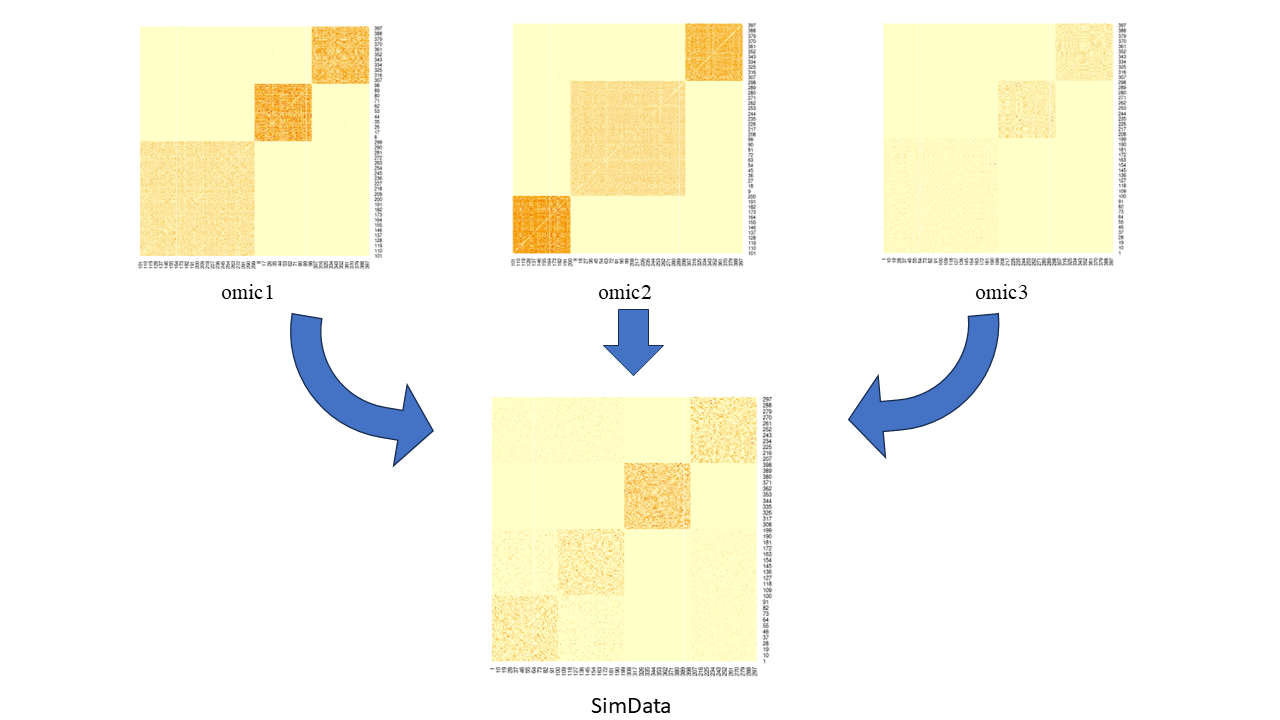


Supplementary Figure 1 Schematic of the synthetic data

**Supplementary Note 3 Permutation Tests**

The permutation test method referenced the approach proposed by Rappoport[2]., during which we randomly shuffled the cluster assignment labels of different samples while keeping the size of each cluster unchanged. For the log-rank test, the number of permutations performed for each clustering solution was initially set as

and continued until the stopping condition was met.

For the clinical enrichment test, we continued performing  permutations until the 95% confidence interval for the -value no longer crossed 0.05, with a maximum of  iterations. This maximum number of iterations was only required when the -value was extremely close to 0.05.

When calculating the exact confidence interval for the -value, we used the binom.test method in R, taking the number of permutations where the chi-square statistic was greater than or equal to that of the original clustering as the number of successes, and the total number of permutations as the number of trials.

References:

[2] Rappoport, N., & Shamir, R. (2018). Multi-omic and multi-view clustering algorithms: review and cancer benchmark. *Nucleic acids research*, *46*(20), 10546–10562. https://doi.org/10.1093/nar/gky889

Supplementary Table 1 Clinical features available for each cancer type.

| Cancer | age | gender | pathologic T | pathologic N | pathologic M | pathologic stage |
| --- | --- | --- | --- | --- | --- | --- |
| AML | √ | √ |  |  |  |  |
| BIC | √ | √ | √ | √ | √ | √ |
| COAD | √ | √ | √ | √ | √ | √ |
| GBM | √ | √ |  |  |  |  |
| KIRC | √ |  | √ | √ | √ |  |
| LIHC | √ | √ | √ | √ | √ | √ |
| LUSC | √ | √ | √ | √ | √ | √ |
| OV | √ |  |  |  |  |  |
| SARC | √ | √ |  |  |  |  |
| SKCM | √ | √ | √ | √ | √ | √ |

Supplementary Table 2 The optimal number of clusters of different methods.

| Cancer | SNF | CIMLR | NEMO | LRAcluster | iClusterBayes | MOSD | MCLS | SCMVC | GDMVC | ULSL |
| --- | --- | --- | --- | --- | --- | --- | --- | --- | --- | --- |
| AML | 4 | 5 | 5 | 3 | 6 | 2 | 5 | 5 | 5 | 5 |
| BIC | 2 | 2 | 3 | 10 | 6 | 2 | 16 | 5 | 2 | 5 |
| COAD | 3 | 3 | 3 | 7 | 6 | 3 | 7 | 4 | 2 | 3 |
| GBM | 2 | 10 | 4 | 6 | 6 | 2 | 7 | 3 | 2 | 5 |
| KIRC | 4 | 6 | 3 | 5 | 6 | 4 | 5 | 4 | 10 | 4 |
| LIHC | 2 | 10 | 5 | 10 | 5 | 4 | 10 | 7 | 7 | 6 |
| LUSC | 2 | 6 | 2 | 8 | 5 | 4 | 12 | 5 | 2 | 3 |
| OV | 3 | 5 | 6 | 6 | 5 | 4 | 13 | 5 | 2 | 4 |
| SARC | 3 | 3 | 3 | 10 | 5 | 3 | 7 | 5 | 2 | 3 |
| SKCM | 3 | 5 | 5 | 6 | 5 | 2 | 12 | 5 | 2 | 5 |


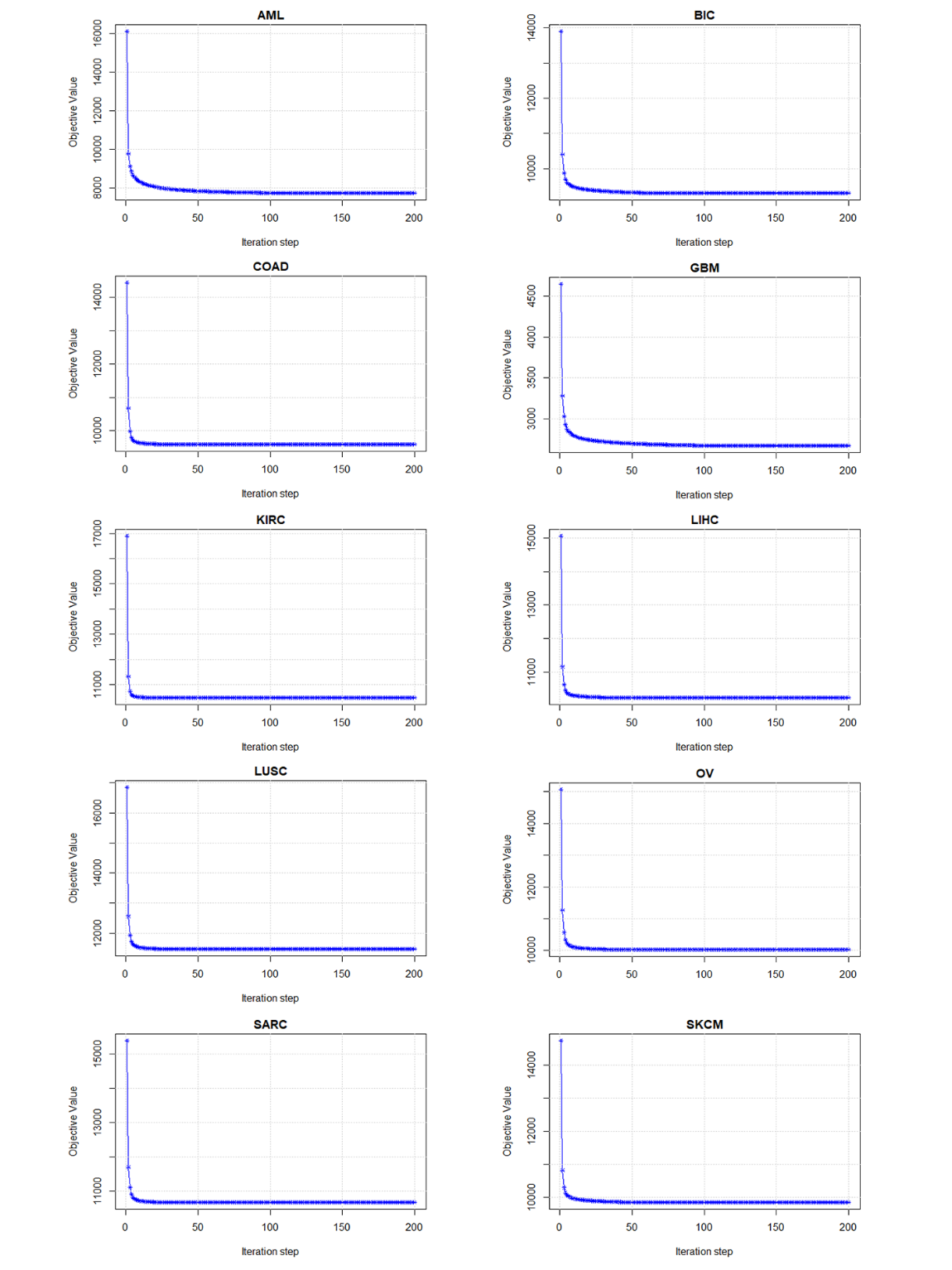


Supplementary Figure 2 Convergence analysis. The objective value decreases with iterations until convergence.


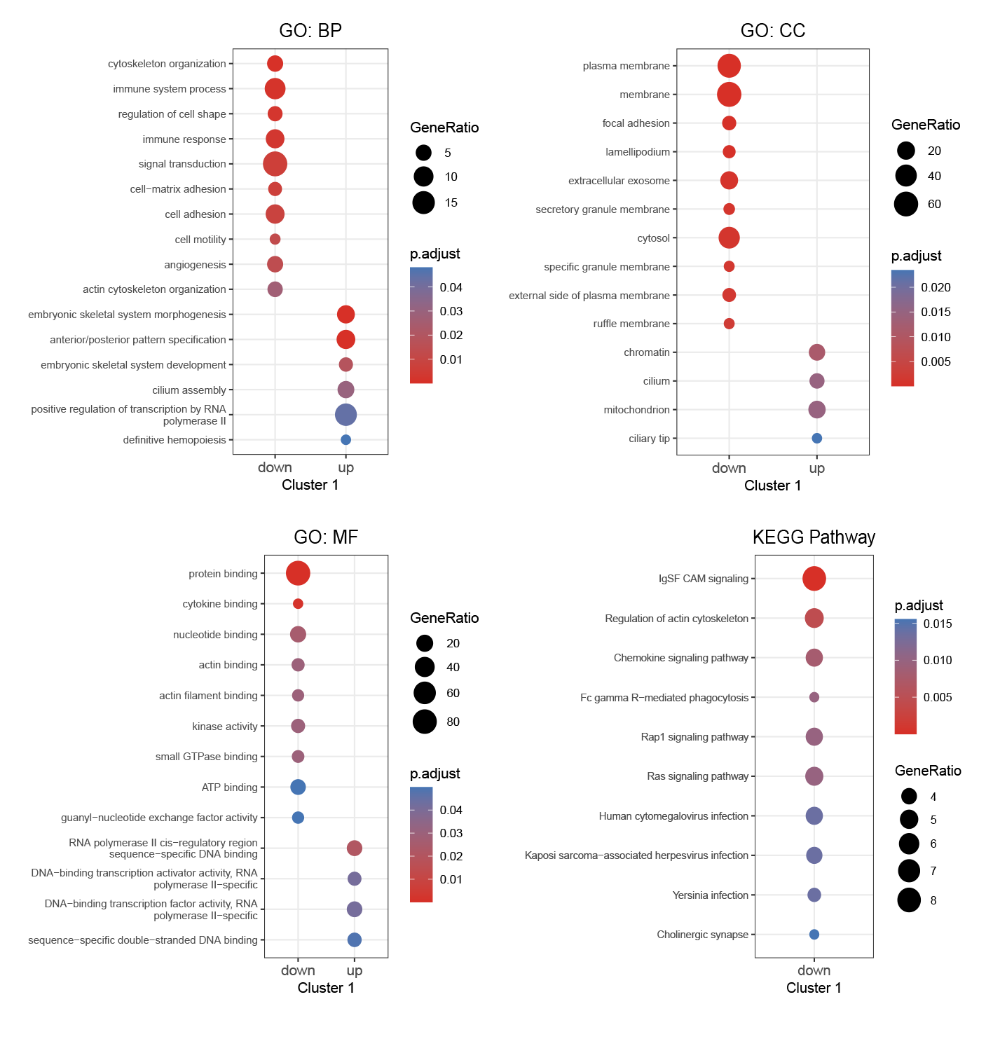


Supplementary Figure 3 GO and KEGG enrichment analysis of AML Cluster 1


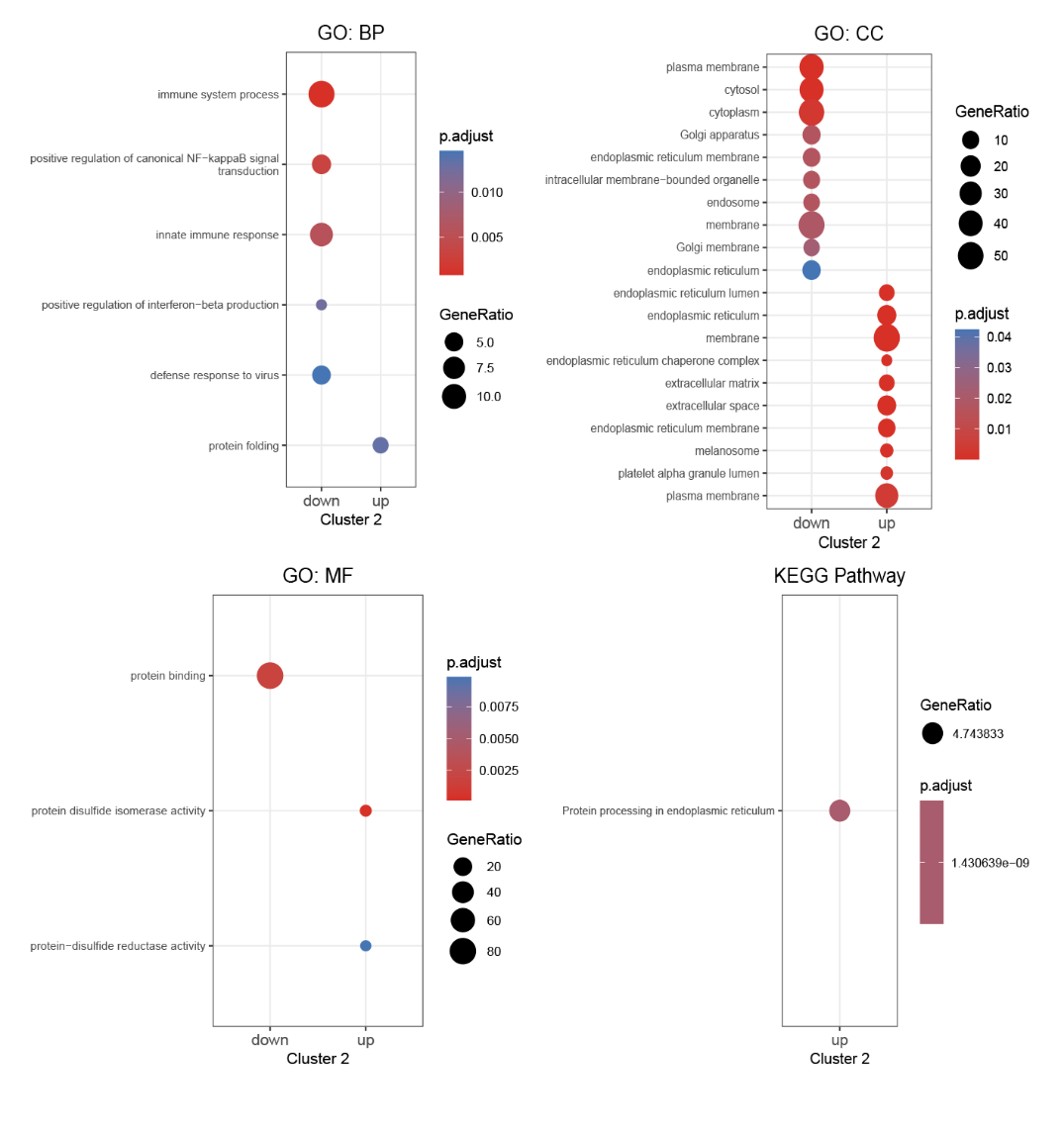


Supplementary Figure 4 GO and KEGG enrichment analysis of AML Cluster 2


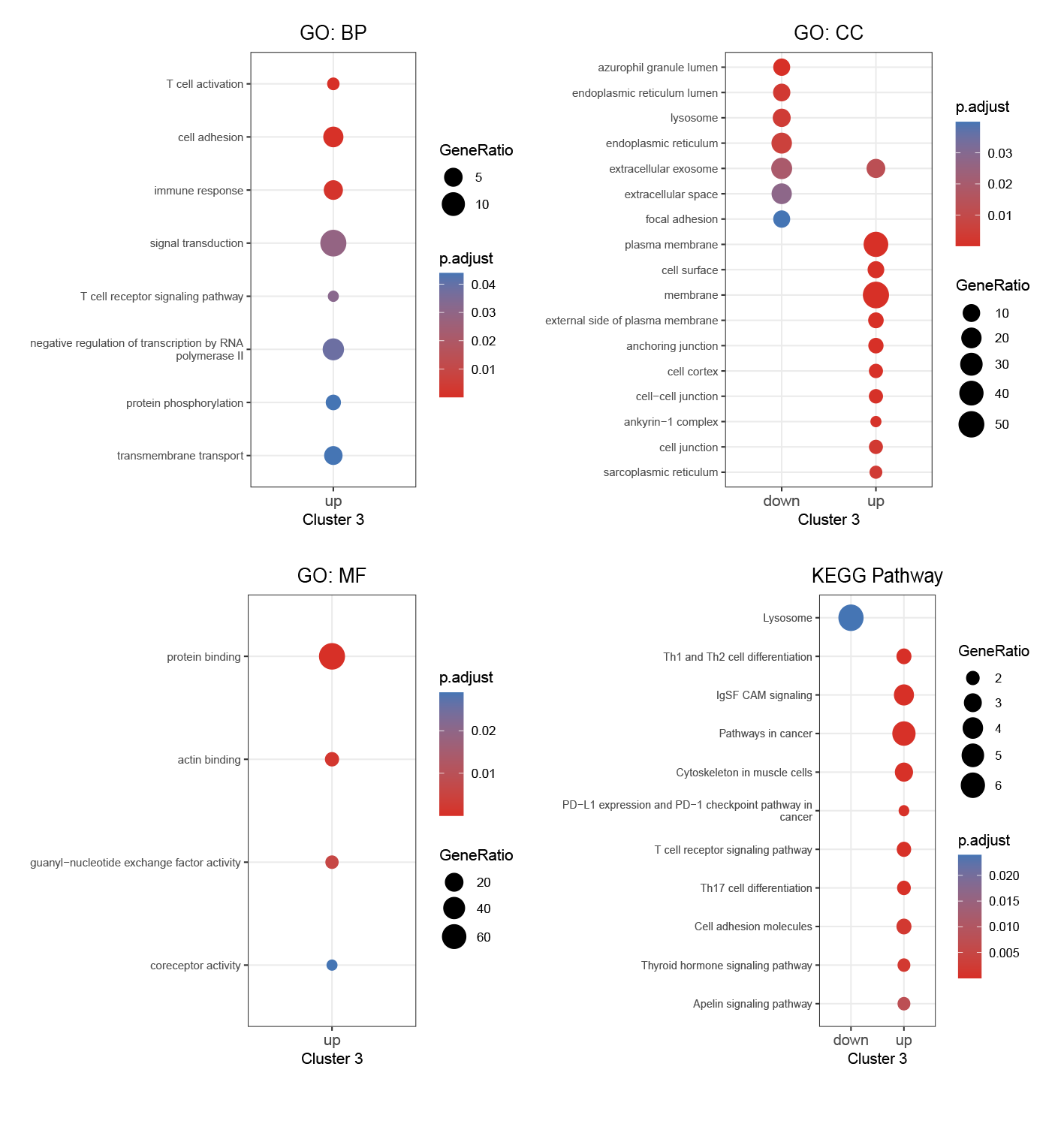


Supplementary Figure 5 GO and KEGG enrichment analysis of AML Cluster 3


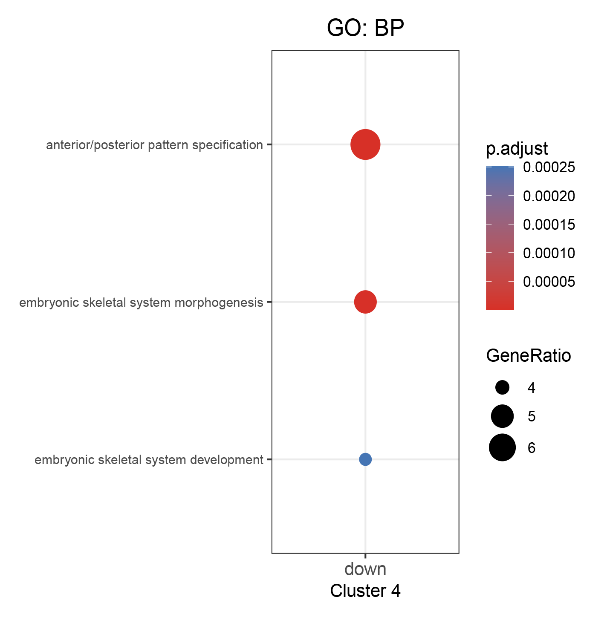


Supplementary Figure 6 GO and KEGG enrichment analysis of AML Cluster 4.The differentially expressed genes in Cluster 4 were exclusively enriched in upregulated Gene Ontology Biological Process (GO-BP) terms.


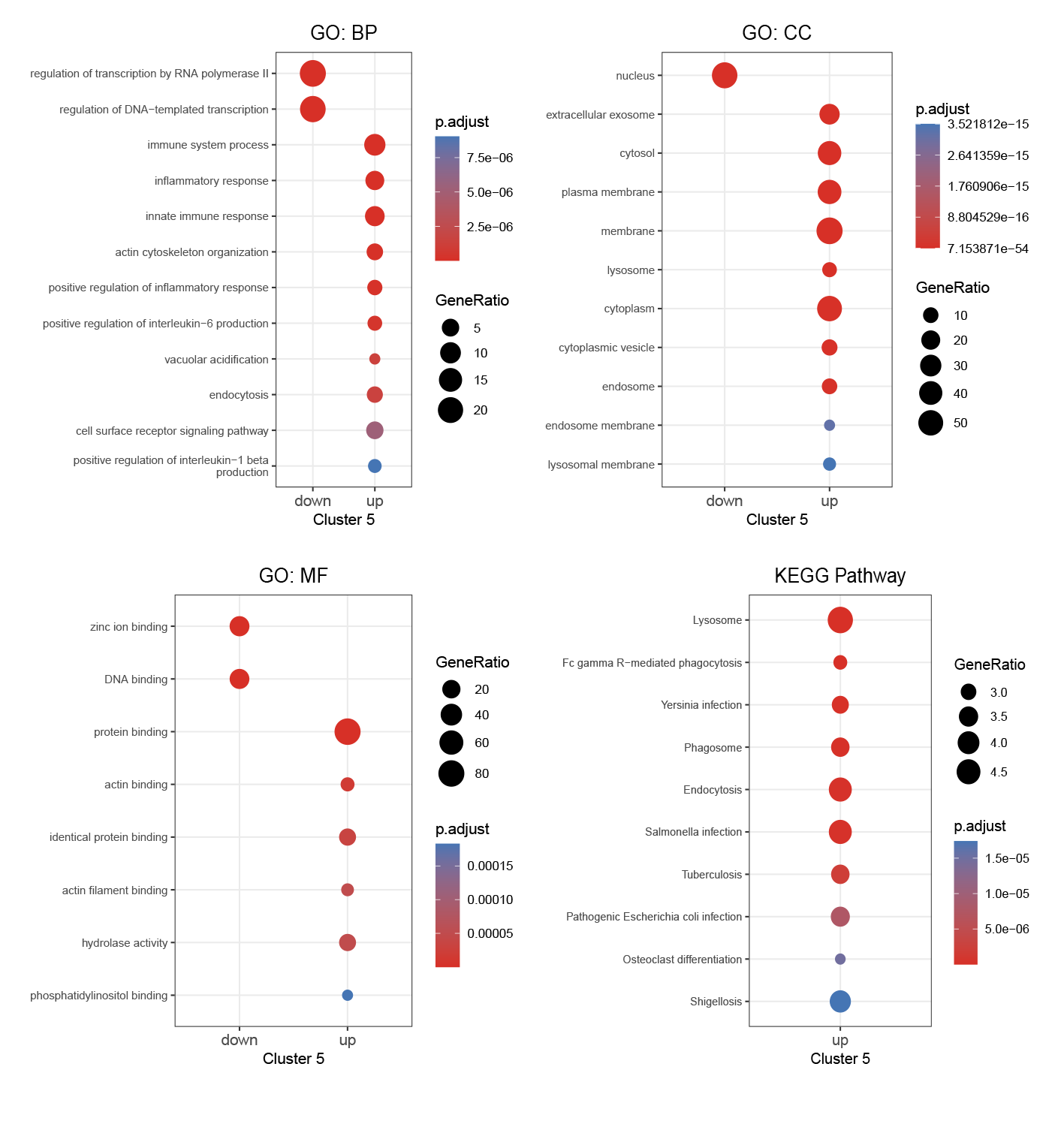


Supplementary Figure 7 GO and KEGG enrichment analysis of AML Cluster 5
